# Pyelonephritis Caused by Multidrug-Resistant Bacteria During Pregnancy: A Case–Control Study

**DOI:** 10.3390/antibiotics15020194

**Published:** 2026-02-10

**Authors:** Gabriel-Ioan Anton, Maria Caliniuc, Carina-Alexandra Bandac, Demetra Gabriela Socolov, Ingrid Andrada Tănasa, Rodica Radu, Radu-Stefan Miftode, Theodor Florin Pantilimonescu, Vlad Ichim, Egidia Gabriela Miftode, Ionela-Larisa Miftode, Viorel Dragoș Radu

**Affiliations:** 1 Department of Obstetrics and Gynecology, Faculty of Medicine, Grigore T. Popa University of Medicine and Pharmacy, 700115 Iasi, Romania; anton.gabriel-ioan@d.umfiasi.ro (G.-I.A.); demetra.socolov@umfiasi.ro (D.G.S.); ingrid-andrada-n-tanasa@d.umfiasi.ro (I.A.T.); 2 Department of Urology, Faculty of Medicine, Grigore T. Popa University of Medicine and Pharmacy, 700115 Iasi, Romania; mg-rom-30762@students.umfiasi.ro (M.C.); carina_bandac@email.umfiasi.ro (C.-A.B.); pantilimonescu.theodor-florin@d.umfiasi.ro (T.F.P.); viorel.radu@umfiasi.ro (V.D.R.); 3 Department of Urology, “Dr. C.I. Parhon” University Hospital, 700503 Iasi, Romania; vlad-gabriel-ichim@students.umfiasi.ro; 4 Department of Internal Medicine, Faculty of Medicine, Grigore T. Popa University of Medicine and Pharmacy, 700115 Iasi, Romania; rodica.radu@umfiasi.ro (R.R.); radu-stefan.miftode@umfiasi.ro (R.-S.M.); 5 Department of Infectious Diseases, Faculty of Medicine, Grigore T. Popa University of Medicine and Pharmacy, 700115 Iasi, Romania; egidia.miftode@umfiasi.ro; 6 Saint Parascheva Clinical Hospital of Infectious Diseases, 700116 Iasi, Romania

**Keywords:** pyelonephritis, pregnancy, multidrug-resistant bacteria, antimicrobial resistance

## Abstract

Background: Pyelonephritis during pregnancy represents a significant maternal–fetal risk, particularly in the context of increasing multidrug-resistant (MDR) bacterial infections. This study aimed to characterize the microbiological profiles and antimicrobial resistance patterns of MDR pathogens causing pyelonephritis in pregnancy. Secondary objectives included the evaluation of patient characteristics, associated risk factors, and pregnancy outcomes. Methods: A retrospective comparative study was conducted including 171 pregnant patients hospitalized with acute pyelonephritis between 1 January 2017 and 30 April 2025. Thirty-four patients with MDR bacterial infections were compared with 137 patients with infections caused by pathogens with conserved antibiotic susceptibility (Non-MDR). Results: Patients with MDR pyelonephritis were significantly older than those with Non-MDR infections (mean age 27.76 vs. 25.30 years, *p* = 0.03). MDR infections were more frequently diagnosed in the third trimester of pregnancy (58.8% vs. 29.9%, *p* = 0.003) and affected multiparous women more often (44.1% vs. 19.7%, *p* = 0.006). No statistically significant differences were observed between groups regarding clinical presentation or laboratory parameters (*p* > 0.05). Prior antibiotic exposure was significantly more common in the MDR group (85.29% vs. 26.61%, *p* < 0.001), as was a history of urological procedures, including urinary catheterization (29.41% vs. 15.10%, *p* = 0.009). For multivariate analysis, two factors were predictive for pyelonephritis with MDR pathogens: previous antibiotic treatment—OR 20.37 (95% CI 2.19–189.88) and urological procedures—OR 13.23 (95% CI 2.24-78-22). *Escherichia coli* was the predominant pathogen in both groups but was isolated more frequently in the Non-MDR cohort (81.75% vs. 58.82%, *p* = 0.015), followed by *Klebsiella pneumoniae*, which appeared more frequently in the study group (23.53% vs. 10.22%, *p* = 0.011). MDR isolates demonstrated significantly higher resistance rates to all tested antibiotics (*p* < 0.05). Complete resistance to ampicillin was observed in the MDR group (100%), compared with 58.01% in the Non-MDR group, indicating markedly reduced efficacy of this agent. Adverse neonatal outcomes were more frequent in the MDR group, with higher rates of Apgar scores < 7 at admission (23.5% vs. 8.8%, *p* = 0.01) and increased neonatal intensive care unit admission (20.6% vs. 7.3%, *p* = 0.02). For multivariate analysis, pyelonephritis with MDR pathogens was predictive for Neonatal Intensive Care Unit (NICU) admission (OR 8.17, 95% CI 2.41–27.67). Conclusions: These findings highlight the need for the periodic revision of empirical antibiotic protocols and risk-adapted therapeutic strategies in pregnant patients in order to reduce maternal and fetal morbidity.

## 1. Introduction

Urinary tract infections (UTIs) during pregnancy are associated with an increased risk of adverse maternal and fetal outcomes [1,2]. Among these, acute pyelonephritis accounts for the majority of complications, including preterm birth, fetal distress, low birth weight, and increased rates of neonatal intensive care unit (NICU) admission [3,4,5]. Pregnancy itself is a well-established risk factor for the development of UTIs [6], including pyelonephritis, due to physiological and anatomical changes specific to gestation [6].

Numerous studies have attempted to identify additional risk factors for the development of pyelonephritis in pregnant women. Some have reported that younger age, nulliparity, and anemia are associated with an increased susceptibility to infection [7,8,9], particularly during the second and third trimesters of pregnancy [8,10]. Among affected patients, those presenting with fever exceeding 38 °C and urinary tract obstruction are at significantly higher risk for developing urosepsis or septic shock [11], conditions that are associated with more than a twofold increase in the risk of preterm birth and a more than fivefold increase in the risk of premature rupture of membranes [12].

The most frequently implicated pathogens in pregnancy-associated pyelonephritis are *Escherichia coli* and *Klebsiella pneumoniae* [13,14]. Earlier studies reported relatively high susceptibility rates of these microorganisms to antibiotics commonly used during pregnancy, such as penicillins and cephalosporins [10,14].

In recent years, however, an increasing number of studies have reported a rising prevalence of UTIs caused by multidrug-resistant (MDR) bacteria [15,16,17,18,19,20], defined as resistance to at least one antimicrobial agent in three or more different antibiotic classes [21]. Nevertheless, most of these studies have evaluated UTIs as a whole, combining cases of cystitis and pyelonephritis within the same study populations [16,17,22], while others did not specifically include pregnant patients [19,20,23].

Only a limited number of studies have focused exclusively on pregnant patients with pyelonephritis caused by MDR bacteria [16,17,18]. These investigations were generally based on small cohorts and primarily addressed patient characteristics and risk factors. Consequently, there is limited global evidence regarding the etiological agents, antimicrobial susceptibility patterns, and specific risk factors associated with acute pyelonephritis due to MDR bacteria in pregnancy. Moreover, pregnancy outcomes have not been adequately evaluated in this particular patient population, as most available studies have assessed the entire spectrum of UTIs—from cystitis and pyelonephritis to urosepsis—without stratified analysis [2,12]. As a result, the true magnitude of adverse maternal and fetal outcomes associated with pyelonephritis with MDR bacteria during pregnancy remains insufficiently understood. Preliminary data from our institution on pyelonephritis caused by MDR pathogens during pregnancy have previously been reported. The present study extends this work by including a larger cohort, a longer observation period, and an expanded analysis of antimicrobial resistance patterns and pregnancy outcomes [24].

In recent years, our institution has encountered an increasing number of cases of acute pyelonephritis caused by MDR pathogens, including among pregnant patients. These cases required particular clinical attention and prolonged follow-up extending beyond delivery. Given the limited and heterogeneous data available in the current literature on this topic, we conducted a retrospective study aimed at characterizing the microbiological profile of the causative pathogens and their antimicrobial resistance patterns, as well as evaluating the maternal and fetal impact of these infections, in comparison with pregnant patients diagnosed with pyelonephritis caused by Non-MDR organisms.

As a secondary objective, we aimed to identify specific risk factors associated with the occurrence of MDR pathogens in pregnant patients and to describe the demographic and clinical characteristics of this subgroup.

## 2. Results

Of the 171 pregnant patients included in the study, 34 were diagnosed with acute pyelonephritis caused by MDR bacteria and constituted the MDR (study) group, while 137 patients with acute pyelonephritis caused by Non-MDR bacteria served as the control group. The baseline demographic and clinical characteristics of patients in the two groups are summarized in Table 1.

No statistically significant differences were observed between the two groups regarding laterality of infection (left vs. right), area of residence (urban vs. rural), or the presence of comorbidities, excepting pregnancy-induced hypertension (8.8% vs. 0%, *p* = 0.0055). Patients in the study group more frequently developed acute pyelonephritis during the third trimester of pregnancy compared with the control group (58.8% vs. 29.9%, *p* = 0.003), whereas pyelonephritis occurred more often during the second trimester among patients in the control group (59.1% vs. 38.2%, *p* = 0.045).

Additionally, patients in the study group were significantly older (*p* = 0.0379) and had a higher proportion of multiparous women, along with a lower proportion of nulliparous patients, compared with the control group (*p* = 0.0062). In both groups, acute pyelonephritis predominantly affected the right kidney, occurred at similar rates among patients from urban and rural areas, and more than 50% of patients were diagnosed with anemia.

No statistically significant differences were observed between the two groups with regard to leukocyte count (*p* = 0.19), serum creatinine levels (*p* = 0.26), or CRP values (*p* = 0.35). Similarly, the presence of fever, sepsis, and septic shock did not differ significantly between groups.

In both cohorts, sepsis was present at hospital admission in approximately one third of cases, and among these patients, nearly one third presented with septic shock. Fever exceeding 38 °C was observed in more than 50% of patients in both groups. No significant differences were identified between the two groups in terms of length of hospital stay (Table 2).

Patients in the study group had a significantly higher prevalence of prior urological procedures in the past 180 days, associated with urinary catheter placement compared with the control group (29.41% vs. 15.10%, *p* = 0.009). Additionally, prior antibiotic exposure within the preceding 180 days was significantly more frequent among patients in the study group, whether prescribed for UTIs or infections at other sites (85.29% vs. 26.61%, *p* < 0.001).

In contrast, patients in the control group more frequently presented with urinary tract obstruction, predominantly gestational hydronephrosis (66.42% vs. 20.58%, *p* < 0.05). In both groups, the indications for ureteral JJ stent placement were gestational hydronephrosis or renoureteral lithiasis. In the study group, all patients diagnosed with hydronephrosis underwent urinary drainage by either JJ stent placement or percutaneous nephrostomy. In the control group, among the 91 patients with hydronephrosis, JJ stents were placed in 82 cases, while 9 patients received conservative management with antibiotic therapy alone due to low-grade hydronephrosis.

In two cases within the study group in which JJ stent insertion was not feasible, percutaneous nephrostomy was performed. At the time of pyelonephritis diagnosis, a significantly higher proportion of patients in the control group presented with gestational hydronephrosis requiring JJ ureteral stent placement compared with the study group (52.55% vs. 26.47%, *p* = 0.004). On multivariate analysis of these factors, only previous antibiotic treatment and history of urological procedures were predictive for development of pyelonephritis with MDR bacteria (OR 20.37, 95% CI 2.19–189.88, *p* = 0.008 and OR 13.23, 95% CI 2.24–78.22, *p* = 0.004). All these data are summarized in Table 3.

The incidence and distribution of bacterial species identified in the two study groups are presented in Table 4. *E. coli* was isolated at a significantly higher rate in the control group compared with the study group (81.75% vs. 58.82%, *p* = 0.015). *K. pneumoniae* was more frequently identified in the study group (23.53% vs. 81.75%, *p* = 0.015). All other bacterial species were detected at comparable frequencies between the two groups.

Notably, coagulase-negative staphylococci were isolated exclusively in the study group; however, these isolates were rare and did not include *Staphylococcus saprophyticus*. Overall, bacteria belonging to the order Enterobacterales represented the predominant etiological agents of pyelonephritis in pregnant patients in both groups. Apart from *E. coli* and *K. pneumoniae*, all other microorganisms were identified at very low frequencies in both cohorts.

The antimicrobial resistance patterns of Enterobacterales isolates identified in the two study groups are summarized in Table 5. Bacterial isolates from the study group exhibited significantly higher resistance rates to all tested antibiotics compared with those from the control group. Resistance to ampicillin reached 100% in the study group, while resistance rates exceeded 50% for trimethoprim–sulfamethoxazole (TMP–SMX), amoxicillin–clavulanic acid, and fluoroquinolones—antibiotics that are commonly prescribed for the treatment of urinary tract infections or other site infections.

In contrast, bacterial isolates largely retained susceptibility to carbapenems and piperacillin–tazobactam, with susceptibility rates of over 90%, agents that are generally considered reserve antibiotics for severe urinary tract infections.

In the control group, bacterial resistance exceeded 50% for ampicillin, while lower resistance rates were observed for TMP–SMX (21.37%) and amoxicillin–clavulanic acid (16.03%), as detailed in Table 5.

Pregnancy and neonatal outcomes are summarized in Table 6. No statistically significant differences were observed between the two groups with respect to overall Apgar scores, birth weight (*p* = 0.12), mode of delivery (cesarean section), or the occurrence of preterm birth and stillbirth.

However, neonates born to mothers diagnosed with pyelonephritis caused by MDR pathogens during pregnancy exhibited significantly higher rates of NICU admission compared with those born to mothers with acute pyelonephritis caused by Non-MDR bacteria (20.6% vs. 7.3%, *p* = 0.02). Additionally, a significantly higher proportion of neonates in the MDR group had Apgar scores below 7 (23.5% vs. 8.8%, *p* = 0.01) and mean Apgar score (7.55 vs. 8.78, *p* = 0.002). On logistic regression analysis, pyelonephritis with MDR pathogens was predictive for NICU admission (OR 8.17, 95% CI 2.41–27.67, *p* = 0.001).

## 3. Discussion

In the present study, we found that pregnant patients may develop acute pyelonephritis caused by MDR pathogens predominantly during the third trimester of pregnancy, most often following prior antibiotic exposure and urological interventions involving urinary drainage devices, such as JJ ureteral stents or percutaneous nephrostomy. These devices were initially placed for gestational hydronephrosis or ureteral lithiasis and were frequently maintained throughout pregnancy. The most commonly identified etiological agents were *E. coli* and *K. pneumoniae*, which exhibited high resistance rates to antibiotics routinely used in the treatment of UTIs, while largely retaining susceptibility to reserve antibiotics such as carbapenems and piperacillin–tazobactam. Importantly, pyelonephritis caused by MDR pathogens during pregnancy was associated with increased rates of NICU admission and a higher proportion of neonates with Apgar scores below 7, without a concomitant increase in other maternal complications when compared with pyelonephritis caused by Non-MDR pathogens.

The mean age of patients in both study groups was approximately 25 years, consistent with previous reports [5,8], supporting the observation that acute pyelonephritis predominantly affects young pregnant women. This finding may be related to limited prenatal health education, delayed presentation for routine antenatal care, or inadequate treatment of cystitis or significant bacteriuria—well-established risk factors for pyelonephritis [3,15,25,26]. However, patients in the MDR group were significantly older and more frequently multiparous, a pattern that may reflect increased exposure to healthcare settings or prior antibiotic treatments, both recognized risk factors for MDR strains.

In both groups, acute pyelonephritis predominantly affected the right kidney, in line with previous studies [5], likely due to the higher incidence of right-sided gestational hydronephrosis, particularly during the second and third trimesters. Our findings confirm that acute pyelonephritis caused by Non-MDR pathogens occurs more frequently during the second trimester [5,10,27], while suggesting that pyelonephritis caused by MDR pathogens tends to develop later in pregnancy, predominantly in the third trimester. This observation may be explained by the presence of urinary drainage devices placed earlier in pregnancy for obstructive pyelonephritis initially caused by Non-MDR bacteria. Urinary catheterization is a well-recognized risk factor for pyelonephritis, particularly infections caused by MDR pathogens [28,29]. Contrary to some reports indicating a higher incidence among nulliparous women [8,27], our study identified a higher prevalence among multiparous patients, consistent with other published data [5].

Regarding comorbidities, anemia was highly prevalent in both groups, affecting more than 50% of patients—a higher rate than reported in some previous studies [7]. While comorbid conditions are known to increase the risk of MDR infections and sepsis, thereby contributing to increased morbidity and mortality [30], few additional comorbidities were observed in our cohort. This finding is not unexpected, as pregnant patients are generally young and otherwise healthy, suggesting that comorbidities likely play a limited role in the development of acute pyelonephritis with MDR bacteria in this population.

Clinical and laboratory findings were comparable between the two groups. Both cohorts exhibited similarly high rates of fever exceeding 38 °C, higher than those reported in some previous studies [31,32], as well as comparable rates of urosepsis and septic shock. These findings suggest that the presence of MDR pathogens does not necessarily increase the risk of sepsis or septic shock in pregnant patients with pyelonephritis, but rather reflects the host immune response, particularly the development of febrile syndrome, as previously described [11]; this contrasts with other reports indicating an increased risk associated with MDR infections [33]. Nonetheless, the overall risk of sepsis in pyelonephritis remains substantial [34], with more than one third of patients in both groups presenting with urosepsis at admission. Although septic shock was more frequent in the MDR group, the difference did not reach statistical significance, possibly due to limited statistical power. Similarly, inflammatory markers such as leukocytosis and CRP did not differ between groups, and MDR pyelonephritis was not associated with increased renal impairment compared with Non-MDR infections, likely reflecting preserved host defense mechanisms despite the potentially increased virulence of MDR bacteria [35].

Analysis of established risk factors for MDR infections revealed that the vast majority of patients in the MDR group had a history of prior antibiotic treatment, with only five cases lacking documented exposure. A substantial proportion of these patients also had a history of urological conditions requiring JJ stent or nephrostomy placement. Notably, the five patients without prior antibiotic exposure had no history of hospitalization or urinary catheterization, supporting the existence of community-acquired MDR pathogens. Thus, MDR infections in pregnancy appear to arise from both healthcare-associated and community-acquired sources [17], highlighting the heterogeneity of this group. Our findings indicate that urological procedures constitute an independent risk factor for MDR infection, but in association with the insertion of urinary catheters, despite previous reports suggesting that JJ stent placement is generally safe and effective [36]. Future studies would be necessary to assess separately previous urological procedures and the presence of urinary catheters as risk factors for MDR infection development. In our cohort, 10 of 24 patients (41.66%) with urinary catheters in both groups developed MDR infections. Both prior antibiotic exposure and urinary catheterization were significantly more frequent in the MDR group, consistent with previous studies [18,19].

Urinary tract obstruction was also evaluated as a potential risk factor. A lower proportion of patients in the MDR group presented with hydronephrosis at admission, indicating that obstruction itself is not a specific risk factor for MDR infection, but rather a general risk factor for obstructive pyelonephritis. However, several patients with MDR pyelonephritis initially developed hydronephrosis requiring urinary drainage, indirectly increasing the risk of catheter-associated MDR infection. Thus, hydronephrosis may contribute indirectly to MDR infection risk through the need for urinary catheterization. In cases of urinary tract obstruction or lithiasis, antibiotic therapy alone may not achieve complete eradication of bacteriuria.

In obstructive pyelonephritis and stone-associated infection, clinical remission after antibiotic therapy does not necessarily imply bacteriological eradication. Urinary tract obstruction and the presence of foreign bodies (e.g., ureteral stents or stones) may act as persistent sources due to impaired drainage and biofilm formation, which can hinder urine sterilization despite appropriate antimicrobial therapy. Current guidance emphasizes that obstruction is a major urological source of urosepsis and that prompt drainage and removal of foreign bodies constitute essential source control measures [37].

Ureteral stents are also prone to bacterial colonization and biofilm, and colonization may occur even when urine cultures are negative, which may contribute to persistent or recurrent bacteriuria [38]. In addition, the close association between urinary stones and bacteria further supports the concept that bacteriological eradication can be challenging when lithiasis is present and often requires combined antimicrobial therapy and urological management [39].

Regarding the bacterial spectrum, a total of eight distinct pathogens were identified in each study group, with seven species shared between the two cohorts, namely *E. coli*, *K. pneumoniae*, *P. aeruginosa*, *P. mirabilis*, *S. marcescens*, *Enterococcus* spp., and *S. aureus*. Although *E. coli* was isolated more frequently in the control group—consistent with its well-established role as the primary etiological agent of UTIs and pyelonephritis [8,10,13,14]—it remained the most prevalent pathogen among cases involving MDR organisms. This observation contrasts with previously reported local data identifying *K. pneumoniae* as the predominant MDR pathogen [40]. In the present study, *K. pneumoniae* represented the second most frequently isolated organism, a finding that may be explained by the inclusion of community-acquired infections rather than exclusively healthcare-associated cases, in which the primary etiologic pathogens are *E. coli*.

In contrast to studies reporting a higher prevalence of *Enterococcus* spp. in UTIs [2,13,23], enterococci were identified at low frequencies in our cohort. However, given the relatively small sample size and the low overall incidence of this pathogen, a higher prevalence cannot be definitively excluded. As anticipated, other members of the Enterobacterales order were detected only sporadically, in accordance with previous reports [11,16].

When comparing antimicrobial resistance patterns among Enterobacterales isolates from the two study groups, we observed—unsurprisingly—a significantly higher resistance rate among isolates from the study group across all tested antibiotics. In line with previous reports [2,22], resistance to ampicillin was very high in both groups, reaching 100% among isolates from the study group. As expected, higher resistance rates in the study group were primarily observed for antibiotics commonly prescribed in the treatment of UTIs, including amoxicillin–clavulanic acid, TMP–SMX, cefuroxime, and fluoroquinolones, findings that are consistent with results from other local studies [11].

Importantly, Enterobacterales isolates largely retained susceptibility to carbapenems, which remain a viable therapeutic option for the treatment of severe pyelonephritis caused by MDR pathogens. This observation underscores the importance of continuous surveillance of local antimicrobial susceptibility patterns when selecting empirical antibiotic therapy [41]. Compared with resistance profiles of Gram-negative bacilli reported in studies conducted in the general population [23,40], MDR isolates identified in pregnant patients in our cohort exhibited a less extensive resistance pattern. This difference may be explained by the relatively limited exposure of pregnant women—who are generally young and without significant comorbidities—to established risk factors for multidrug resistance. Although fosfomycin represents an emerging therapeutic option during pregnancy, susceptibility data were not consistently available in our cohort and were therefore not included in the analysis.

The antimicrobial resistance patterns observed in this cohort should be interpreted within the broader context of local and regional epidemiology. In recent years, our institution has documented a progressive increase in resistance among Enterobacterales to commonly prescribed antibiotics, including aminopenicillins, beta-lactam/beta-lactamase inhibitor combinations, third-generation cephalosporins, and fluoroquinolones, in both secondary care and tertiary referral settings.

Local Romanian studies have documented high antimicrobial resistance among urinary pathogens. For example, *Escherichia coli* isolates from female patients showed significant resistance to fluoroquinolones and other commonly used agents [42]. Recent work during the COVID-19 pandemic reported increasing proportions of ESBL-producing *E. coli* and *Klebsiella pneumoniae*, suggesting escalating AMR pressure in UTIs [43]. Broader surveillance of Gram-negative pathogens in Romania has further shown that antibiotic resistance genes and MDR Enterobacterales are widely disseminated in clinical and environmental settings [44]. Together, these data highlight the local antimicrobial resistance epidemiology against which our findings on MDR pyelonephritis in pregnancy should be interpreted, supporting the need for tailored empiric therapy guided by regional resistance patterns.

An important objective of the present study was to evaluate the maternal and neonatal impact of pyelonephritis caused by MDR pathogens. No maternal deaths were recorded; however, two neonatal deaths occurred during the study period. Although statistical analysis did not reveal significant differences between groups, it remains possible that MDR-associated pyelonephritis increases the risk of fetal distress and neonatal mortality compared with Non-MDR infections. The absence of statistically significant findings may be attributable to the relatively small sample size. Notably, differences were observed with respect to NICU admission rates and Apgar scores. It is plausible that concerns regarding neonatal infection risk and lower Apgar scores prompted neonatologists to proceed with NICU admission to ensure closer monitoring and early detection of neonatal distress [2].

Although pyelonephritis during pregnancy is a well-established risk factor for adverse outcomes such as low birth weight, preterm delivery, premature rupture of membranes, and fetal distress [1,2,6], the presence of MDR pathogens did not confer an additional risk for these outcomes in our cohort. This finding may be explained by the structure of prenatal care in our country, where pregnant women benefit from dedicated monitoring, free access to medical services, and national healthcare programs that facilitate early diagnosis and prompt initiation of appropriate treatment, including antibiotic therapy.

In our clinical practice, empirical treatment of acute pyelonephritis during pregnancy typically consists of beta-lactam antibiotics, such as aminopenicillins or second- or third-generation cephalosporins, in accordance with local guidelines. However, the high resistance rates observed among MDR isolates to these agents suggest that, in patients with identifiable risk factors—such as recent antibiotic exposure or prior urological procedures—early escalation to broader-spectrum agents, including piperacillin–tazobactam or carbapenems, may be warranted until culture results become available.

The present study focused exclusively on symptomatic acute pyelonephritis and does not address the management of asymptomatic bacteriuria caused by MDR pathogens during pregnancy. In such cases, therapeutic options may be broader and include agents such as fosfomycin, nitrofurantoin, or pivmecillinam, provided that renal function is preserved. Management strategies for asymptomatic MDR bacteriuria should therefore be considered separately from those of acute upper UTI.

To our knowledge, this is the first study to specifically focus on pregnant patients with acute pyelonephritis caused by MDR pathogens and to systematically assess maternal and fetal outcomes in comparison with a control group of patients with Non-MDR infections. Nevertheless, several limitations should be acknowledged. The retrospective design and the relatively small sample size—reflecting the rarity of this condition—may have limited the statistical power to detect subtle differences between groups and to establish definitive causal relationships. The low annual incidence of MDR pyelonephritis also precluded a prospective study design. Follow-up until delivery was not feasible for all patients, as some delivered in obstetric units other than the study center; additionally, the exclusion of patients without complete microbiological or follow-up data may have introduced selection bias and could limit generalizability.

We also acknowledge that lower bacterial count thresholds are currently accepted for the diagnosis of urinary tract infections; therefore, the use of a ≥10^5^ CFU/mL cutoff may have led to the underestimation of early or low-count infections, particularly in mild cases. Despite its single-center design, the extended study period and increased sample size strengthen the analysis and support the robustness of the observed associations. Future prospective, multicenter studies involving larger cohorts are warranted to validate these findings. The results of this study are of particular relevance to urologists, obstetricians–gynecologists, and infectious disease specialists, as they may aid in early diagnosis, informed antibiotic selection, and appropriate maternal–fetal surveillance throughout pregnancy and the postpartum period.

## 4. Materials and Methods

We conducted a retrospective case–control study including pregnant patients diagnosed with acute pyelonephritis who were hospitalized in the Urology and Nephrology Departments of the “Dr. C.I. Parhon” University Clinical Hospital between 1 January 2017 and 30 April 2025. Patients were subsequently followed at the “Cuza Vodă” University Hospital, the largest tertiary referral center for obstetrics and gynecology in eastern Romania. A subset of patients overlapped with a previously published study from our institution; however, the current analysis includes additional cases, a longer study period, and expanded outcome and resistance analyses.

Pregnant patients were admitted to either the urology or nephrology department of the “Dr. C.I. Parhon” Hospital according to clinical presentation. As a general institutional practice, patients with pyelonephritis associated with urinary tract obstruction were admitted to the urology department, whereas those without associated obstruction were managed in the nephrology department.

The study protocol was approved by the Ethics Committee of the “Dr. C.I. Parhon” University Clinical Hospital (approval no. 4703/15 May 2025) and by the Ethics Committee of the “Cuza Vodă” University Hospital (approval no. 23/23 October 2025). Patient data were extracted from the hospitals’ electronic medical records using ICD-10 codes from the International Classification of Diseases, 10th Revision [45]. All pregnant patients hospitalized during the study period were considered eligible for inclusion.

Initially, pregnant patients admitted to the two departments of the “Dr. C.I. Parhon” Hospital during the study period were identified using the ICD-10 code O09 (supervision of high-risk pregnancy), resulting in a total of 282 patients. Of these, 31 patients without documented urinary tract infection (including cases of urolithiasis, gestational hydronephrosis, and chronic kidney disease), 6 patients diagnosed with cystitis, and 27 patients with a diagnosis of pyelonephritis but without a documented urine culture in the electronic records were excluded.

Among the remaining 218 patients, 47 could not be identified in the electronic medical records of the “Cuza Vodă” University Hospital and were therefore excluded. Consequently, a total of 171 pregnant patients with documented pyelonephritis were included in the final analysis. These patients were subsequently divided into two study groups based on the presence or absence of a positive urine culture with MDR pathogens.

**Figure 1:**
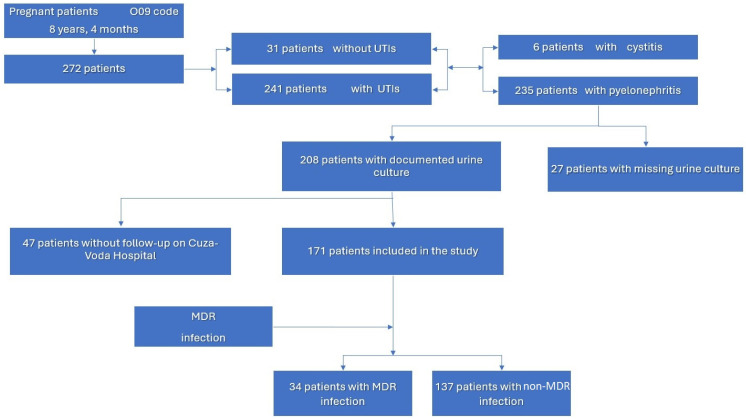
Patient selection process; UTIs—Urinary tract infections; MDR—multidrug resistant.

The patient selection process is illustrated in Figure 1.

Patient characteristics were extracted from the electronic medical records of the “Dr. C.I. Parhon” University Clinical Hospital and included age, laterality of infection (left or right), gestational trimester at the time of pyelonephritis diagnosis, area of residence (urban or rural), parity, and the presence of comorbidities. Clinical and laboratory data collected at admission included leukocyte count, C-reactive protein (CRP) levels, serum creatinine values, and the presence of fever, urosepsis, or septic shock.

Potential risk factors analyzed comprised the presence of urinary tract obstruction, prior antibiotic exposure within the preceding 180 days, history of urological procedures in the past 180 days, and urinary catheterization. Microbiological data included identification of bacterial isolates and analysis of their antimicrobial susceptibility profiles in relation to antibiotics commonly used in routine clinical practice. Maternal and fetal outcomes were evaluated by assessing birth weight, Apgar score, NICU admissions, occurrence of preterm birth, and evidence of fetal distress.

A positive urine culture was defined as bacterial growth exceeding 100,000 colony-forming units per milliliter (CFUs/mL). Antimicrobial susceptibility testing was initially performed using the disk diffusion method and, where applicable, broth microdilution, with interpretation based on the European Committee on Antimicrobial Susceptibility Testing (EUCAST) clinical breakpoint tables for minimal inhibitory concentrations (MICs) and inhibition zone diameters, as well as EUCAST guidance on expected resistance phenotypes. Results were interpreted using the EUCAST breakpoint versions in force at the time of isolate identification: version 7.1 for isolates identified in 2017; versions 8.0 (until 16 May 2018) and 8.1 (from 17 May 2018) for isolates identified in 2018; version 9.0 for 2019; version 10.0 for 2020; version 11.0 for 2021; version 12.0 for 2022; version 13.1 for 2023, including the June 2023 update introducing breakpoints for anaerobic bacteria; version 14.0 for 2024; and version 15.0 for isolates identified between 1 January and 30 April 2025 [46]. The following antibiotic discs (MAST Group Ltd., Mast House, Bootle, UK) were used: ampicillin (10 µg), amoxicillin–clavulanic acid (10–20 µg), cefuroxime (30 µg), ceftriaxone (30 µg), ceftazidime (10 µg), cefepime (30 µg), piperacillin–tazobactam (6–30 µg), ciprofloxacin (5 µg), levofloxacin (5 µg), imipenem (10 µg), meropenem (10 µg), gentamicin (10 µg), fosfomycin (200 µg), nitrofurantoin (100 µg), and trimethoprim–sulfamethoxazole (1.25–23.75 µg). Antimicrobial susceptibility testing was performed using the MicroScan WalkAway DxM1040 automated system (Beckman Coulter, Indianapolis, IN, USA).

Urine samples were collected from all patients within the first hour after hospital admission, prior to the initiation of empirical antibiotic therapy. Each urine culture was processed and interpreted by one of two experienced microbiologists in our institution.

The diagnosis of sepsis and septic shock was established according to the Sepsis-3 criteria [47]. Anemia was defined as a hemoglobin level below 11 g/dL. Gestational age was categorized into trimesters as follows: first trimester (weeks 1–13), second trimester (weeks 14–27), and third trimester (weeks 28–40).

The diagnosis of acute pyelonephritis was based on the presence of clinical and laboratory features consistent with upper UTI, including febrile syndrome, flank or lumbar pain, and leukocytosis, in association with a positive urine culture. No cases of renal or perirenal abscess were identified in the study population. Imaging studies, primarily renal ultrasound, were performed to all patients at admittance, to detect the presence of hydronephrosis.

Antibiotic therapy was individualized according to the severity of each case and the presence of risk factors for MDR pathogens. Given that a substantial proportion of patients presented with sepsis or septic shock at admission, antibiotic treatment was administered primarily intravenously. In patients without identified risk factors for MDR infection, empirical therapy most commonly consisted of beta-lactam antibiotics, including third-generation cephalosporins. In severe cases or in patients with known risk factors for MDR pathogens, empirical treatment was initiated with carbapenems. Following availability of antimicrobial susceptibility testing results, antibiotic regimens were adjusted accordingly. The duration of therapy was individualized based on clinical response and disease severity.

### Statistical Analysis

Quantitative variables were summarized using means and standard deviations, while qualitative variables were expressed as frequencies and percentages. Data normality was assessed using the Kolmogorov–Smirnov test. Comparisons between groups of quantitative variables were performed using the Student’s *t*-test for normally distributed data. Categorical variables were compared using the chi-square test or Fisher’s exact test, as appropriate, when expected cell counts were less than five. For multivariate analysis, we utilized multivariable logistic regression.

Statistical analyses were initially performed using Microsoft Excel and subsequently transferred to IBM SPSS Statistics software, version 22 (IBM Corp., Armonk, NY, USA). A *p*-value < 0.05 was considered statistically significant.

## 5. Conclusions

Our findings indicate that the most frequently identified etiological agents of pyelonephritis caused by MDR pathogens during pregnancy are *E. coli* and *K. pneumoniae*. These organisms exhibit increasing resistance to commonly used antibiotics, while largely retaining susceptibility to carbapenems. Prior antibiotic exposure and previous urological interventions involving prolonged urinary catheterization emerged as the main risk factors associated with MDR infections in pregnant patients.

Acute pyelonephritis caused by MDR pathogens was associated with higher rates of NICU admission and lower Apgar scores at birth. These findings underscore the importance of the continuous adaptation of antibiotic therapy guidelines for pregnant patients based on local antimicrobial resistance patterns, as well as the early initiation of appropriate antibiotic treatment in order to minimize complications, particularly those affecting the fetus.

## Figures and Tables

**Table 1:** Characteristics and Demographic Data of MDR and Non-MDR Groups.

	MDR (*n* = 34)	Non-MDR (*n* = 137)	*p*-Value
Age (years), mean ± SD	27.76 ± 6.02	25.30 ± 6.11	0.0379
Infection site, *n* (%)			0.95
Right	23 (67.64%)	94 (68.6%)	
Left	7 (20.58%)	30 (21.8%)	
Bilateral	4 (11.76%)	13 (9.4%)	
Trimester of pregnancy, *n* (%)			0.0055
1st	1 (2.9%)	15 (10.9%)	
2nd	13 (38.2%)	81 (59.1%)	
3rd	20 (58.8%)	41 (29.9%)	
Place of origin, *n* (%)			0.8819
Urban	13 (38.2%)	48 (35.0%)	
Rural	21 (61.8%)	89 (65.0%)	
Parity, *n* (%)			0.0062
Nullipara	19 (55.9%)	110 (80.3%)	
Parity ≥ 1 (Multipara)	15 (44.1%)	27 (19.7%)	
Comorbidities, *n* (%)			
Anemia	20 (58.82%)	75 (54.7%)	0.5406
Diabetes mellitus	2 (5.9%)	1 (0.7%)	0.1873
Pregnancy-induced hypertension	3 (8.8%)	0 (0.0%)	0.0055
Sinus tachycardia	1 (2.9%)	2 (1.5%)	1.0000
Thrombophilia	2 (5.9%)	1 (0.7%)	0.1873
Thyroiditis	0 (0.0%)	3 (2.2%)	0.8880
Chronic kidney disease	0 (0.0%)	1 (0.7%)	1.0000

**Table 2:** Clinical and paraclinical data in MDR vs. non-MDR patients.

	MDR (*n* = 34)	Non-MDR (*n* = 137)	OR (95% CI)	*p*-Value
Clinical data, *n* (%)				
Sepsis	11 (32.35%)	49 (35.8%)	3.29 (1.50–7.22)	0.84
Septic shock	5 (14.7%)	9 (6.47%)	6.25 (2.45–15.92)	0.16
Fever ≥ 38 °C	19 (55.8%)	58 (42.6%)	2.17 (1.01–4.70)	0.07
Paraclinical data, mean ± SD				
Leukocytosis (cells/mm^3^)	14,530 ± 4789	16,120 ± 11,245	-	0.19
CRP (mg/L)	98.48 ± 84.41	114.36 ± 94.08	-	0.3468
Creatinine (mg/dL)	0.81 ± 0.87	0.64 ± 0.40	-	0.2612
Hospitalization days	7.12 ± 8.20	4.66 ± 1.63	-	0.0963

**Table 3:** Potential risk factors associated with MDR vs. non-MDR patients.

	MDR (*n* = 34)	Non-MDR (*n* = 137)	OR (95% CI)	*p*-Value
Double-J catheter insertion	9 (26.47%)	81 (59.85%)	3.08 (1.34–7.08)	0.005
Nephrostomy catheter insertion	2 (5.9%)	0 (0.0%)	21.15 (0.99–451.34)	0.0495
Gestational hydronephrosis	9 (26.47%)	91 (66.42%)	5.5 (2.37–12.73)	0.0001
Reno/ureteral lithiasis	4 (11.76%)	20 (14.38%)	1.28 (0.38–4.35)	0.63
History of urological procedures (past 180 days)	10 (29.41%)	14 (15.1%)	3.66 (1.41–9.60)	0.035
Previous antibiotic treatment (past 180 days)	29 (85.29%)	37 (26.61%)	15.7 (5.6–44.3)	0.0001

**Table 4:** Incidence and type of bacteria in MDR vs. non-MDR patients.

Microorganism	MDR (*n* = 34)	Non-MDR (*n* = 137)	*p*-Value
*Escherichia coli n* (%)	20 (58.82%)	112 (81.75%)	0.015 ^C^
*Klebsiella pneumoniae n* (%)	8 (23.53%)	14 (10.22%)	0.011 ^C^
*Proteus mirabilis n* (%)	1 (2.94%)	2 (1.46%)	0.548 ^F^
*Serratia marcescens n* (%)	1 (2.94%)	1 (0.73%)	0.287 ^F^
*Staphylococcus aureus n* (%)	1 (2.94%)	1 (0.73%)	0.287 ^F^
*Pseudomonas aeruginosa n* (%)	1 (2.94%)	2 (1.46%)	0.548 ^F^
*Enterococcus* spp. *n* (%)	1 (2.94%)	5 (3.64%)	1 ^F^
Coagulase-negative staphylococci *n* (%)	1 (2.94%)	0 (0.0%)	0.199 ^F^

^C^, Chi-square test; ^F^, Fisher’s exact test.

**Table 5:** Antibiotic resistance patterns of Enterobacterales isolates in MDR vs. non-MDR patients.

Antibiotic	MDR (*n* = 31)	Non-MDR (*n* = 131)	*p*-Value
Ampicillin	31 (100.00%)	76 (58.01%)	<0.05 ^c^
Nitrofurantoin	6 (19.35%)	5 (3.81%)	<0.05 ^c^
Trimethoprim–sulfamethoxazole	26 (83.87%)	28 (21.37%)	<0.05 ^c^
Amoxicillin–clavulanic acid	17 (54.83%)	21 (16.03%)	<0.05 ^c^
Piperacillin–tazobactam	3 (9.67%)	0 (0.00%)	0.009 ^F^
Imipenem	1 (3.2%)	0 (0.00%)	0.19 ^F^
Meropenem	1 (3.2%)	0 (0.00%)	0.19 ^F^
Cefuroxime	24 (77.41%)	2 (1.52%)	<0.05 ^F^
Ceftriaxone	12 (38.70%)	0 (0.00%)	<0.001 ^F^
Ceftazidime	12 (38.70%)	0 (0.00%)	<0.001 ^F^
Cefepime	10 (32.25%)	0 (0.00%)	<0.001 ^F^
Ciprofloxacin	27 (87.09%)	3 (2.29%)	<0.001 ^F^
Levofloxacin	21 (67.74%)	2 (1.52%)	<0.001 ^F^
Gentamicin	13 (41.93%)	0 (0.00%)	<0.001 ^F^

^C^, Chi-square test; ^F^, Fisher’s exact test.

**Table 6:** Pregnancy and neonatal outcomes in MDR vs. non-MDR groups.

Outcome	MDR (*n* = 34)	Non-MDR (*n* = 137)	*p*-Value
Birth weight, g (mean ± SD)	2950 ± 520	3100 ± 480	0.120
Apgar score ≤ 7, *n* (%)	8 (23.5%)	12 (8.8%)	0.010
Apgar score (mean ± SD)	7.55 ± 2.14	8.78 ± 1.11	0.002
Preterm birth (<37 weeks), *n* (%)	5 (14.70%)	9 (6.47%)	0.157
Cesarean birth, *n* (%)	20 (58.8%)	60 (43.8%)	0.090
Neonatal NICU admission, *n* (%)	7 (20.6%)	10 (7.3%)	0.020
Stillbirth, *n* (%)	2 (5.88%)	1 (0.7%)	0.12

## Data Availability

The original contributions presented in this study are included in the article. Further inquiries can be directed to the corresponding author.
